# Bmserpin2 Is Involved in BmNPV Infection by Suppressing Melanization in *Bombyx mori*

**DOI:** 10.3390/insects10110399

**Published:** 2019-11-11

**Authors:** Shahzad Toufeeq, Jie Wang, Shang-Zhi Zhang, Bing Li, Pei Hu, Lin-Bao Zhu, Ling-Ling You, Jia-Ping Xu

**Affiliations:** 1 School of Life Sciences, Anhui Agricultural University, Hefei 230036, China; toufeeqphd@ahau.edu.cn (S.T.); wangjie_3001@163.com (J.W.); 18755148780@163.com (S.-Z.Z.); libing2504@sina.com (B.L.); m15755074091@163.com (P.H.); zhulinbao@163.com (L.-B.Z.); 13063306714@163.com (L.-L.Y.); 2 Anhui International Joint Research and Developmental Center of Sericulture Resources Utilization, Hefei 230036, China

**Keywords:** *Bombyx mori*, NPV, Serpin2, melanization

## Abstract

Melanization, an important defense response, plays a vital role in arthropod immunity. It is mediated by serine proteases (SPs) that convert the inactive prophenoloxidase (PPO) to active phenoloxidase (PO) and is tightly regulated by serine protease inhibitors (serpins) which belong to a well distributed superfamily in invertebrates, participating in immune mechanisms and other important physiological processes. Here, we investigated the *Bmserpin2* gene which was identified from a transcriptome database in response to *Bombyx mori* nucleopolyhedrovirus (BmNPV) infection. Quantitative real-time polymerase chain reaction (qRT-PCR) results showed that *Bmserpin2* was expressed in all tissues, with maximum expression in fat body. Upon BmNPV infection, the expression of *Bmserpin2* was up-regulated in P50 (susceptible strain) and BC9 (resistant strain) in haemocytes, fat body and the midgut. However, up-regulation was delayed in BC9 (48 or 72 h), in contrast to P50 (24 h), after BmNPV infection. Meanwhile, Bmserpin2 could delay or inhibit melanization in silkworm haemolymph. Significant increased PO activity can be observed in *Bmserpin2*-depleted haemolymph under NPV infection. Furthermore, the viral genomic DNA copy number was decreased in *Bmserpin2*-depleted haemolymph. We conclude that *Bmserpin2* is an inducible gene which might be involved in the regulation of PPO activation and suppressed melanization, and have a potential role in the innate immune system of *B. mori*.

## 1. Introduction

*Bombyx mori* is a well-known lepidopteran insect with a great economic value as a producer of silk. *B. mori* is also used in many studies as model insect in genetics and applied biotechnology [1]. *Bombyx mori* nucleopolyhedrovirus (BmNPV) is a major burden for silkworms that causes serious loss to the sericulture industry. BmNPV contains two types of virion phenotypes, budded virus (BV) and occlusion-derived virus (ODV). BV transfects cell-to-cell, while ODV spreads from one host to another host [2]. Most *B. mori* strains are highly susceptible to BmNPV, while only a few are highly resistant [3]. Studies on susceptible and resistant strains have increased the understanding of the mechanisms triggered by virus infection, however, a detailed understanding of *B. mori* resistance to BmNPV infection is yet elusive [4].

Insects solely depend on their innate immunity which comprises of humoral and cellular responses to combat pathogens [5,6]. Besides humoral and cellular responses, intracellular responses, such as apoptosis, RNAi and melanization also contribute to insect defenses [7]. Melanization is an important immune component in the insect defence system and is stimulated by the serine proteases (SPs) cascade that converts inactive prophenoloxidase (PPO) to active phenoloxidase (PO). This leads to the synthesis of quinones and melanin which encapsulates the invading pathogens [8,9,10]. For successful elimination of pathogens, expression of serine proteases (SPs), and their activation is tightly regulated by serine protease inhibitors (serpins), which are the largest known superfamily of protease inhibitors in vertebrates and invertebrates. A typical serpin structure contains a serpin domain and a 20-amino-acid reactive centre loop (RCL) at the C-terminus, acting as “bait” for target proteases [11]. During the inhibition process, serpin interacts with its target protease, and uses its RCL to bait the protease and go through dramatic conformational change which eventually inhibit the protease [12,13].

Insect serpins are key players in the defense mechanism of insects, especially the Toll pathway and PPO cascade [14]. Due to their crucial role in insect immunity, serpins have been widely investigated in several insects that are model organisms and/or agricultural pests, including *Drosophila melanogaster* [15], *Bombyx mori* [16], *Manduca sexta* [17], *Helicoverpa armigera* [18], *Tenebrio molitor* [19], and *Tribolium castaneum* [20]. Biochemical studies revealed that serpins are negative regulators of PPO. For example, in *M. sexta* serpin-1J, serpin-3, serpin-6, serpin-7 and serpin-12 negatively regulate PPO cascade via inhibiting proteases [8,21,22,23]. Several serpins including serpin-5, serpin-6, serpin-15, serpin-18, and serpin-32 from *Bombyx mori* have proved to negatively affect the PPO pathway [24,25,26,27,28].

Recent studies have suggested that melanization is involved in combating virus infection in larval insects. For instance, in tobacco budworm, haemolymph acts as a viricide [29]. Moreover, 5,6-dihydroxyindole (DHI), a melanin precursor, has broad-spectrum antiviral activity [30]. Also, the PO cascade in *Aedes aegypti* blocked Semliki Forest virus (SFV) infection [31]. Yuan et al. revealed that serpin-5 and serpin-9 regulate melanization and promote baculovirus infection [32]. However, there have been very few studies on silkworm serpins in response to BmNPV infection.

To better understand the silkworm serpins and melanization mechanism, we studied *B. mori* serpin-2 (Bmserpin2) under BmNPV infection. We found that *Bmserpin2* can be induced by BmNPV infection, and knockdown of serpin-2 increases PO activity. This study should support further study on the serpins in response to BmNPV infection.

## 2. Materials and Methods

### 2.1. Rearing of Silkworm and B. mori Nucleopolyhedrovirus (BmNPV) Preparation

The *B. mori* susceptible strain (P50) and resistant strain (BC9) were preserved in the Anhui International Joint Research and Development Centre of Sericulture Resources Utilization, Anhui Agricultural University, Hefei, China. BC9 is a near-isogenic strain which was obtained when P50 and A35 (a highly resistant strain to BmNPV) were crossed, and offspring were repeatedly backcrossed with P50 for nine generations to construct BC9, and each offspring was screened for BmNPV [33]. Larvae were reared using fresh mulberry leaves at 26 ± 1 °C, 75 ± 5% relative humidity with 12 h day/night cycles. The BmNPV T3 strain was maintained and purified as described previously [34].

### 2.2. BmNPV Infection to B. mori

Virus infection was carried out according to previous published study [35]. Briefly, P50 and BC9 (3rd day fifth instar) larvae were fed orally with 5 µL OBs (1 × 10^6^ OBs/mL in water), while each larva in the control group was administrated with 5 µL of water. Normal feeding continued after infection. The insects were sacrificed, and different tissues (haemocytes, fat body and the midgut) were collected at different time points after infection. The samples were frozen in liquid N_2_ and stored at −80 °C for later use.

### 2.3. Total RNA Isolation and Amplification of BmSerpin2

Total RNA from silkworm tissues was extracted by Trizol reagent (Takara, Dalian, China), and first-strand cDNA was synthesised using the PrimeScript RT kit (TaKaRa, Dalian, China) according to the manufacturer’s instructions. *Bmserpin2* was identified from a previous study [33], and gene specific primers (Table 1) were designed with the Primer premier 5.0 software and used to amplify the *Bmserpin2* open reading frame (ORF). A polymerase chain reaction (PCR) was carried out as follows: pre-denaturation at 94 °C for 5 min; 35 cycles at 94 °C for 30 s, 55 °C for 30 s, and 72 °C for 1 min 20 s and 72 °C for 10 min for extension. The PCR products were resolved on 1% agarose gel and purified using a DNA Purification Kit (Tiangen Biotech, Beijing, China). Purified PCR products were cloned into the pMD19-T vector and sequenced to verify the cloned fragments.

### 2.4. Recombinant Bmserpin2 and Antibody Preparation

The cloned cDNA fragment that contained mature Bmserpin2 ligated into the expression vector pET-28a (Novagen) with restriction enzyme sites *BamH1* and *Xho1*, and the insertion was confirmed by DNA sequencing in the recombinant BmSerpin2-pET-28a plasmid and then the recombinant plasmid was transformed into *Escherichia coli* BL21 (Novagen) competent cells which were grown in fresh Luria-Bertani (LB) medium. The positive plasmid was induced by isopropyl β-D-thiogalactoside (IPTG) (1 mM) to induce recombinant Bmserpin2 expression for 6 h at 37 °C. After centrifugation at 7500× *g* for 10 min at 4 °C, bacteria were collected and sonicated in phosphate-buffered saline (PBS). The insoluble pellet was dissolved in buffer A (100 mM NaH_2_PO_4_, 10 mM Tris-HCl, 8 M urea, pH 8.0). Bmserpin2 protein was purified using a Ni-NTA Fast Start Kit (Qiagen, Inc., Valencia, CA, USA) according to the manufacturer’s protocol. After purification, the protein was analysed by 12% sodium dodecyl sulfate polyacrylamide gel electrophoresis (SDS-PAGE) and western blotting using anti-His-antibody. The concentration of Bmserpin2 protein was determined by using the BCA protein Assay Kit (Novagen, Hilden, Germany). The polyclonal antibody of Bmserpin2 was prepared by HuaAn Biotechnology Co., Ltd. (Hangzhou, China).

### 2.5. Preparation of Soluble Bmserpin2

To perform the biochemical analysis of Bmserpin2, the purified recombinant Bmserpin2 was renatured through a dialysis method. 3 mL of Bmserpin2 (0.2 mg/mL) was loaded into the dialysis bag (Sangon Biotech, Shanghai, China). Bmserpin2 was dialysed in the refolding buffer (5% glycerol, 1% arginine, 2% glycine and the following urea gradient: 6 M, 4 M, 3 M, 2 M, 1 M, 0 M) and rotated on ice from high to low urea gradients for 2 h per gradient. After dialysis, the protein was centrifuged at 12,000× *g* for 10 min at 4 °C. The supernatant was collected, and ultrafiltration was carried out using centrifugal filter devices (Millipore, Bedford, MA, USA). The protein was saved and used for later experiments.

### 2.6. Expression Analysis of Bmserpin2 in Different Tissues and Developmental Stages

Total RNA was extracted from different tissues (midgut, fat body, haemocytes, integument, Malpighian tubules, head and silk gland) and developmental stages (egg to adult) using Trizol reagent (Invitrogen, Grand Island, NY, USA) according to the manufacturer’s instructions. The concentration and integrity of RNA were observed at an absorbance ratio of A_260/280_ and A_260/230_ using a NanoDrop 2000 spectrophotometer (Thermo Scientific^TM^, Waltham, MA, USA), and by 1.0% agarose gel electrophoresis, respectively. Total RNA samples were used to prepare first strand cDNA using PrimeScript^TM^ RT Master Mix (Takara, Dalian, China) following the manufacturer’s instructions. The qRT-PCR was performed with a TB Green kit (Takara) and analyzed with the Multicolour Real-time PCR Detection System (Bio-Rad, Hercules, CA, USA), as previously reported [36]. The thermal cycling profile was set as follows: initial denaturation at 95 °C for 30 s and 40 cycles of 95 °C for 5 s, 60 °C for 30 s, and 72 °C for 20 s. The transcriptional level of *Bmserpin2* was normalized to *B. mori GAPDH*. The relative expression levels were calculated using the 2^−∆∆Ct^ method according to a previous protocol [37]. The experiment was conducted in triplicate.

### 2.7. Expression Analysis of Bmserpin2 after BmNPV Infection

To determine the expression of *Bmserpin2* after BmNPV infection, different tissues including haemocytes, fat body and the midgut were collected from P50 and BC9 strains at 6, 12, 24, 48 and 72 h after infection. Total RNA was extracted and cDNA was prepared as described above. The mRNA level of *Bmserpin2* was analysed by qRT-PCR. The transcriptional level of *Bmserpin2* was normalized to *B. mori GAPDH*. The experiment was conducted in triplicate.

### 2.8. Western Blotting

Proteins were extracted from the midgut at 24 h post infection (hpi) and haemolymph (24, 48 and 72 hpi) of P50 and BC9 strains. 1 mL of protein lysis buffer (4% CHAPS, 2 M thiourea, 7 M urea), 0.12 g samples of midgut or 500 μL samples of haemolymph, 1 mM phenyl methane sulfonyl fluoride (PMSF) and 10 mg of dithiothreitol (DTT) were added into glass homogenisers for homogenising. After centrifugation at 12,000× *g* at 4 °C for 40 min, protein concentrations were determined by BCA. The extracted protein (25 µg) were loaded on SDS-PAGE and transferred to polyvinylidene fluoride (PVDF) membrane, and incubated with the anti-Bmserpin2 (1:500) primary antibody. GAPDH antibody (1:1000) was used as a reference. Horseradish peroxidase (HRP)-conjugated goat anti-rabbit and goat anti-mouse (Transgene Biotech, Beijing, China) (1:5000 dilution) were used as secondary antibodies.

### 2.9. Effect of Bmserpin2 on the Melanization of Haemolymph

To observe the effect of Bmserpin2 on the melanization of the haemolymph, a previous protocol was followed with a few modifications [28]. Briefly, the cell-free haemolymph from the 3rd day fifth instar larvae was collected. The sample (5 µL) was incubated with 0, 1, 2, 4 and 6 µg of soluble prepared Bmserpin2 diluted in PBS. The reaction was performed at room temperature and captured by camera to observe colour difference in samples at 0, 5, 10 and 20 min.

### 2.10. Synthesis and Micro-Injection of Small Interfering RNA (siRNA)

RNAi experiment was performed by following previous protocol [38]. In short, siRNA sequences were obtained from two different regions of the *Bmserpin2* gene using an online tool (http://bioinfo.clontech.com/rnaidesigner/sirnaSequenceDesignInit.do). The siRNAs were synthesised by GenePharma Technology Co. Ltd. (Shanghai, China). Table 2 shows the siRNA sequences. The siRNAs were injected to 3rd day of 5th instar larvae. Each larva of the siRNA group was injected with 2 µL (2 µg/µL) of siRNA solution. Larvae in the control group were injected with same dose of si green fluorescent protein (siGFP) as control. After 24 h of the injection of siRNAs, larvae were infected with BmNPV for downstream experiments. The experiment was conducted in triplicate.

### 2.11. Phenoloxidase (PO) Activity Assay

The PO activity assay was conducted using a previously described protocol [32]. Briefly, cell-free haemolymph samples were collected from P50 at 48 h after BmNPV infection without adding phenylthiourea (PTU). Samples were incubated at 27 °C for 30 min and the PO activity assay was conducted in 96-well plates containing 1 µL haemolymph followed by the addition of 200 µL substrate solution (2 mM dopamine in 50 mM sodium phosphate buffer (pH 6.5). PO activity was measured at 490 nm in a microtiter plate reader. One unit of PO activity was defined as Δ490 of 0.001 in one minute. The PO activity of siRNA treated cell-free haemolymph of *Bmserpin2* were performed in the same way as described above.

### 2.12. Examination of Viral DNA in Haemolymph

The virus genomic DNA from *Bmserpin2* depleted haemolymph was extracted by following previous protocols [39]. Briefly, haemolymph (100 µL) was mixed with 100 µL of 1 M NaOH, and incubated at room temperature for 5 min, then 20 µL of ammonium acetate was added and samples were incubated again for 5 min. An equal volume of phenol/chloroform (1:1) was used for extraction and samples were centrifuged at 12,000× *g* for 10 min. The supernatant was collected and precipitated in absolute ethanol. The final pellet was resuspended in 20 µL TE buffer. qRT-PCR was carried out to measure the viral genomic DNA copies using BmNPV *GP64*. *B. mori GAPDH* was used to normalize the expression of target gene. The assay was performed in triplicate.

## 3. Results

### 3.1. Tissue Distribution and Expression of *BmSerpin2* Gene at Different Developmental Stages

**Figure 1:**
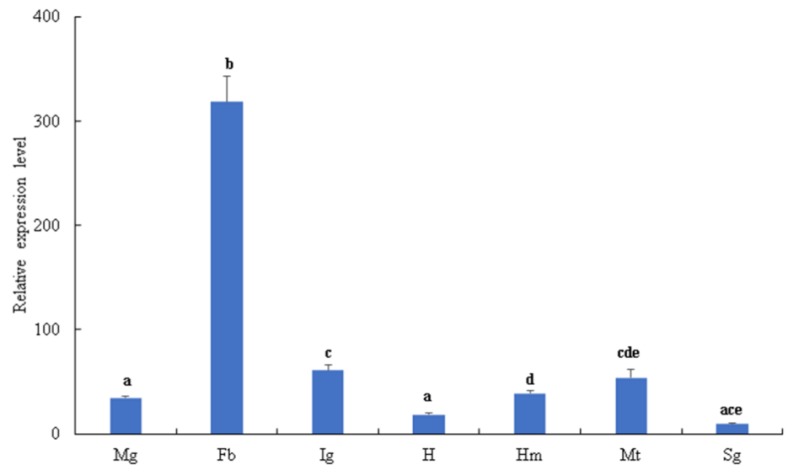
Tissue distribution of *Bmserpin2* in the 5th instar *B. mori* larvae. The mRNA levels of *Bmserpin2* were analysed by qRT-PCR. The *Bmserpin2* transcript level in the midgut was used as the calibrator. Data were normalised with *Bombyx mori* glyceraldehyde 3-phosphate dehydrogenase (GAPDH). Error bars show mean ± standard deviation (SD, *n* = 3). Significant difference is indicated with different letters (one-way ANOV followed by Tukey’s test, *p* < 0.05). Mg: Midgut, Fb: Fat body, Ig: Integument, H: Head, Hm: Haemolymph, Mt: Malpighian tubule, Sg: Silk gland.

**Figure 2:**
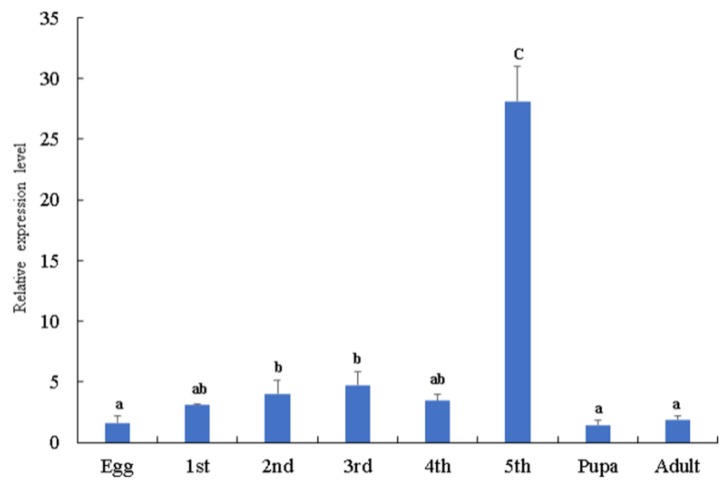
The expression pattern of *Bmserpin2* in different developmental stages using whole insect body. Analysis of mRNA levels of *Bmserpin2* in different developmental stages using qRT-PCR. The *Bmserpin2* transcript level in the egg was used as the calibrator. Data were normalised with *Bombyx mori* GAPDH. Error bars show mean ± SD (*n* = 3). Significant difference is indicated with different letters (one-way ANOVA followed by Tukey’s test, *p* < 0.05).

The tissue distribution and developmental stages of *Bmserpin2* expression levels were analysed by qRT-PCR. The results indicated that *Bmserpin2* was transcribed in all tissues including the midgut, fat body, haemocytes, integument, Malpighian tubules and head and silk gland. However, the transcriptional level of *Bmserpin2* was markedly higher in the fat body, and the lowest expression was observed in the silk gland (Figure 1). Furthermore, the developmental stages result revealed that the expression level of *Bmserpin2* was the highest in fifth instar larvae, and the expression in larvae was higher than in pupae and adults (Figure 2).

### 3.2. Expression Analysis of Bmserpin2 in Response to BmNPV Infection

**Figure 3:**
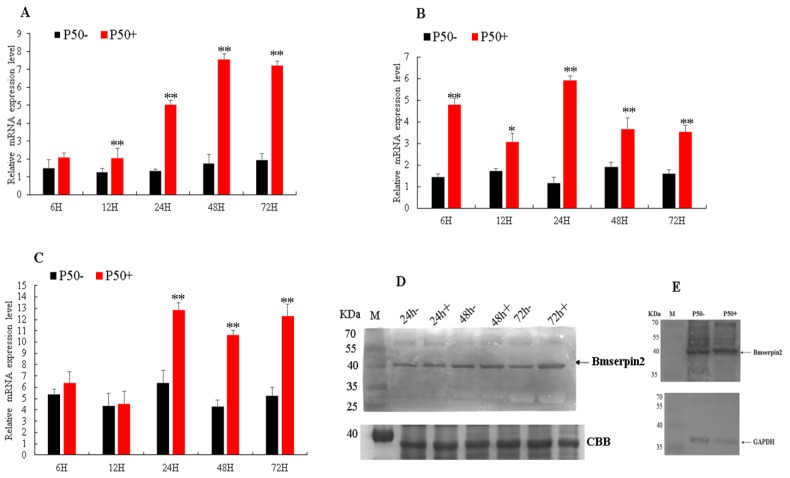
Expression profiles of the *Bmserpin2* in P50 (susceptible strain) after *Bombyx mori* nucleopolyhedrovirus (BmNPV) infection in the haemocytes (**A**), fat body (**B**) and midgut (**C**) at 6, 12, 24, 48 and 72 hpi. P50− was used as a control. Relative expression levels were calculated using the 2^−∆∆Ct^ method. Statistical analysis was performed using SPSS software (Version 19.0, Armonk, NY, USA). The significant differences are indicated by * (*p* < 0.05) or ** (*p* < 0.01). Western blot analyses were performed in haemolymph (**D**) at 24, 48 and 72 hpi, total proteins were coomassie brilliant blue (CBB)-stained as the loading control and in midgut (**E**) at 24 hpi. Anti-GAPDH was used as the loading control.

To investigate the mRNA expression level of *Bmserpin2* in response to BmNPV infection, silkworm P50 and BC9 strains were used. Temporal expression was analysed by qRT-PCR in the haemocytes, fat body and midgut. Our results suggested that compared with P50− (control), *Bmserpin2* mRNA expression in P50+ (infected) was significantly increased after 24, 48 and 72 hpi in the haemocytes (Figure 3A). In the fat body, *Bmserpin2* expression varied after post-infection, however, its expression was highest at 24 h (Figure 3B). The relative expression of *Bmserpin2* in the midgut increased significantly at 24 h, and its expression was significant until 72 hpi (Figure 3C). Protein levels of Bmserpin2 in the haemolymph and midgut were upregulated after BmNPV infection (Figure 3D,E).

**Figure 4:**
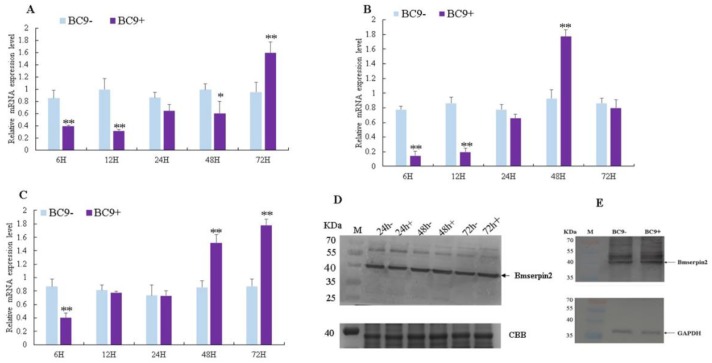
Expression profiles of the *Bmserpin2* in BC9 (resistant strain) after BmNPV infection in the haemolymph (**A**), fat body (**B**) and midgut (**C**) at 6, 12, 24, 48 and 72 hpi. BC9− was used as a control. Relative expression levels were calculated using the 2^−∆∆Ct^ method. Statistical analysis was performed using SPSS software. Significant differences are indicated by * (*p* < 0.05) or ** (*p* < 0.01). Western blot analyses were performed in haemolymph (**D**) 24, 48 and 72 hpi, total proteins were Coomassie Brilliant Blue (CBB)-stained as the loading control and midgut (**E**) at 24 hpi. Anti-GAPDH was used as the loading control.

*Bmserpin2* expression in BC9 after BmNPV infection revealed that its expression, compared to BC9− (control), was downregulated in BC9+ (infected) from 6 to 48 h, however, its expression was significantly induced at 72 h in haemocytes (Figure 4A). The *Bmserpin2* expression in the fat body was downregulated at the beginning (6–12 h) but increased gradually from 24 h and was significantly upregulated at 48 h (Figure 4B). In the midgut, *Bmserpin2* was downregulated until 24 h, then significantly upregulated at 48 and 72 hpi (Figure 4C). Furthermore, Western blot analyses indicated that protein levels of Bmserpin2 in the haemolymph and midgut of the BC9 strain were upregulated after BmNPV infection (Figure 4D,E). Therefore, the above results suggest that Bmserpin2 expression was induced in response to BmNPV infection and might play an important role in *B. mori* after BmNPV infection.

### 3.3. Bmserpin2 Inhibits Melanization in Haemolymph

**Figure 5:**
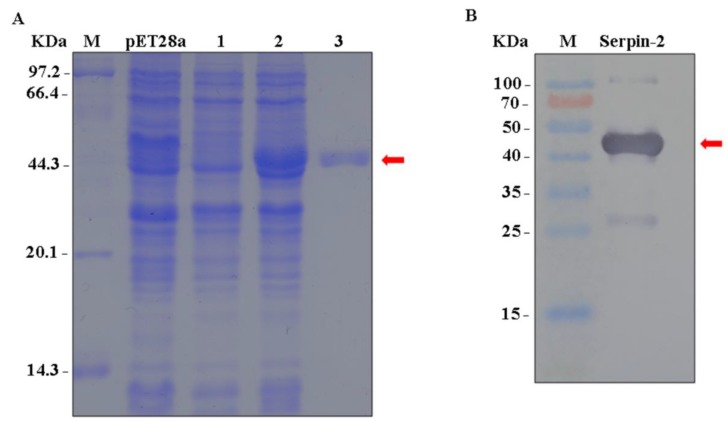
(**A**) Analysis of recombinant Bmserpin2 protein SDS-PAGE. M: molecular weight markers. pET-28a used as a control. Lane 1: Negative control without induction. Lane 2: Induced expression under isopropyl β-D-thiogalactoside (IPTG). Lane 3: Purified recombinant Bmserpin2 protein. (**B**) Western blot analysis of recombinant Bmserpin2 using anti-His antibody.

The recombinant His-tagged *Bmserpin2* was expressed with pET-28a in *E. coli* (BL21) competent cells. Expressed *Bmserpin2* protein was successfully detected by SDS-PAGE with a molecular weight of 44.3 kDa (Figure 5A). Western blotting was employed using anti-His antibodies which confirmed the target protein of 44.3 kDa (Figure 5B).

**Figure 6:**
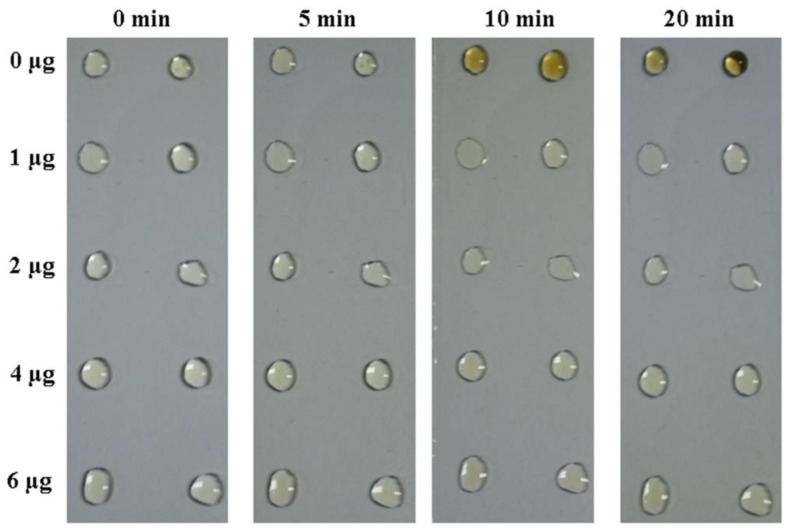
Bmserpin2 inhibits melanization in silkworm haemolymph. Cell-free haemolymph was mixed with different amounts of recombinant Bmserpin2. The control was prepared without Bmserpin2. The change in colour of haemolymph was observed and photographed.

To investigate the effect of Bmserpin2 on haemolymph melanization, cell-free haemolymph from P50 larvae was incubated with biologically active Bmserpin2. Haemolymph was exposed to air to observe spontaneous melanization. The colour of the haemolymph sample in the control group slowly became dark brown within 20 min. However, haemolymph mixed with Bmserpin2 (1–6 µg) showed a decrease in melanization. This suggested that Bmserpin2 could inhibit the melanization in *B. mori* (Figure 6).

### 3.4. Silencing of Bmserpin2 in B. mori Increased PO Activity in Response to BmNPV Infection

**Figure 7:**
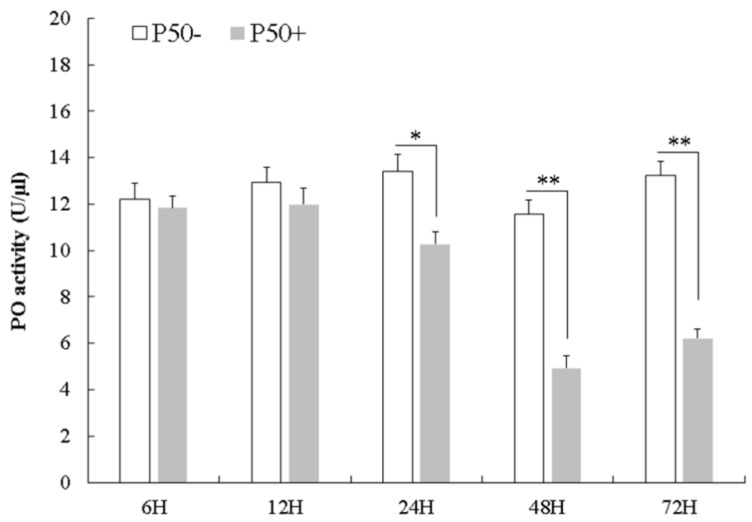
Phenoloxidase (PO) activity of cell-free haemolymph in control and infected *B. mori* at 6, 12, 24, 48 and 72 hpi. Error bars show mean ± SD (*n* = 3). Statistical analysis was performed using SPSS software. Significant differences are indicated by * (*p* < 0.05) or ** (*p* < 0.01).

**Figure 8:**
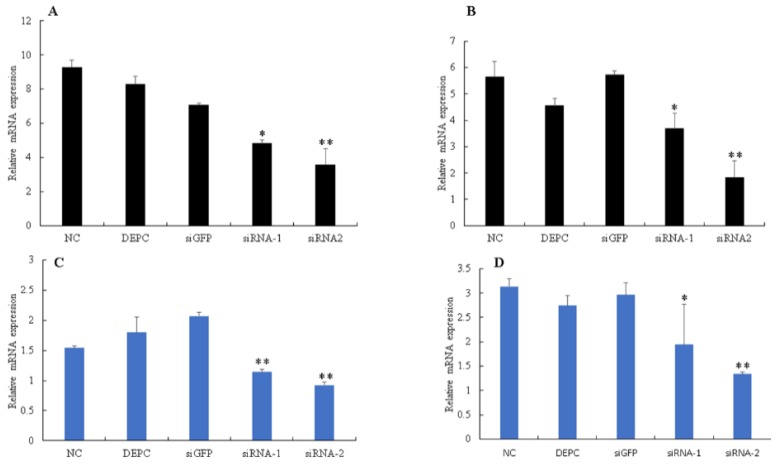
RNAi efficiency estimation of *Bmserpin2*. The expression level of *Bmserpin2* in haemocytes and the fat body was measured at 24 h and 48 h. Haemocytes (**A**,**B**); Fat body (**C**,**D**) after injection of siRNAs by qRT-PCR. NC: Negative control, DEPC: diethyl pyrocarbonate (DEPC) water, siGFP: si green fluorescent protein, siRNA-1: first target sequence of Bmserpin2 knockdown, siRNA-2: Second target sequence of Bmserpin2 knockdown. Statistical analysis was performed using SPSS software. Significant differences are indicated by * (*p* < 0.05) or ** (*p* < 0.01).

**Figure 9:**
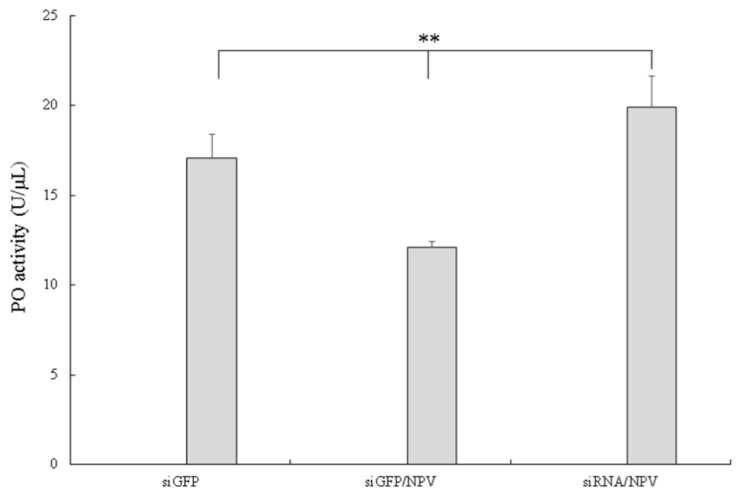
Knockdown of *Bmserpin2* increased PO activity in cell-free haemolymph. PO activity was assayed using dopamine as a substrate, as described in “Materials and methods”. Haemolymph collected from GFP (siGFP), GFP infected with BmNPV (siGFP/NPV) and Bmserpin2 siRNA (siRNA2 target sequence) infected with BmNPV (siRNA/NPV) was used to measure PO activity. Error bars show mean ± SD (*n* = 3). Statistical analysis was performed using SPSS software. Significant differences are indicated by * (*p* < 0.05) or ** (*p* < 0.01).

To further investigate melanization in silkworms after BmNPV infection, PO activity was assessed in cell-free haemolymph at 6, 12, 24, 48 and 72 hpi using the P50 strain. The results showed that PO activity in infected plasma did not show any changes at 6 and 12 hpi, however, as infection progressed, PO activity started to decrease at 24 hpi, and decreased remarkably at 48 and 72 hpi as compared to control (Figure 7). To explore the effect of *Bmserpin2* on PO activity, we conducted knockdown of *Bmserpin2* by injecting siRNA into 3rd day of fifth instar larvae. RNAi efficiency was detected by qPCR at 24 and 48 h after siRNA injection in haemocytes and fat body tissues, and indicated the transcript level of *Bmserpin2* was greatly decreased (Figure 8). After 24 h injection of siRNA, cell-free haemolymph was collected from larvae and PO activity was measured. We found that knockdown of *Bmserpin2* showed remarkable increase in PO activity than control after BmNPV infection (Figure 9). These results showed that Bmserpin2 could inhibit melanization by regulating PO activation.

### 3.5. Virus Genomic DNA Copies Decreased in BmSerpin2-Depleted Haemolymph

**Figure 10:**
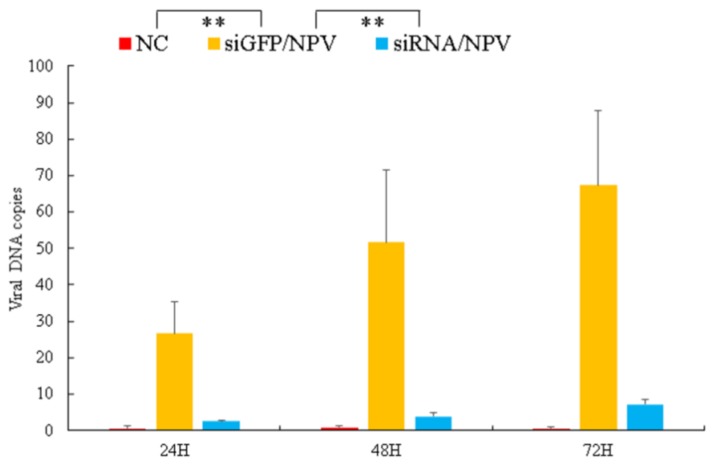
Viral genomic copies in *Bmserpin2*-depleted haemolymph. The BmNPV viral DNAs were extracted from haemolymph, and qPCR was performed to determine viral genomic DNA copies. Haemolymph from negative control (NC), GFP infected with BmNPV (siGFP/NPV) and Bmserpin2 siRNA infected with BmNPV (siRNA/NPV) were collected at 24, 48 and 72 h after BmNPV infection. Data were shown as mean ± SD (*n* = 3). Statistical analysis was performed using SPSS software. Significant differences are indicated by * (*p* < 0.05) or ** (*p* < 0.01).

As knockdown of *Bmserpin2* increased the PO activity after BmNPV infection, we next analysed virus genomic DNA copies in haemolymph after knockdown of *Bmserpin2* at 24, 48 and 72 h post BmNPV infection. The results revealed that viral DNA copies were significantly reduced in contrast to siGFP (Figure 10). These results indicated that *Bmserpin2* might be important for BmNPV to overcome host melanization.

## 4. Discussion

Serpins belong to the largest superfamily of serine protease inhibitors with wide range of physiological functions in insects. Serpins are involved in innate immunity by inhibiting the serine protease cascade that triggers melanization [14]. In previous research, many studies were conducted on serpins from *B. mori* in response to bacterial challenges [24,26,28]. We identified *Bmserpin2* from the transcriptome, which showed different expression in different resistant silkworm strains [33]. However, the role of *B. mori* serpins in response to BmNPV had not been conducted extensively.

Several studies have shown that serpins are expressed differentially in different tissues and developmental stages [16,22,24]. In the present study, we observed the expression of *Bmserpin2* in different tissues, and its expression was highest in the fat body. A similar expression of serpins has been characterised in different organisms, where it is also mainly expressed in the fat body which is responsible for production of most haemolymph proteins [23,26,40]. Meanwhile, we noted expression of *Bmserpin2* in developmental stages, with it being highly expressed in 5th instar larvae. Likewise, Yang et al. noted an increased transcriptional level of serpin-12 in 5th instar larvae of *M. sexta* [23]. The fat body is an important tissue involved in innate immunity and a source of haemolymph protein synthesis [41], and the development of resistance in silkworms increases as the larvae grows. Thus, these results suggest that *Bmerpin2* may participate in the innate immunity of the silkworm.

The mRNA expression level of *Bmserpin2* in P50 and BC9 significantly increased in the haemocytes, fat body and midgut after BmNPV infection, and protein levels of Bmserpin2 were also increased in the haemolymph and midgut post BmNPV infection. For P50 strain, *Bmserpin2* was upregulated at 24, 48 and 72 h after infection in the haemocytes and midgut; however, its expression started to upregulate at 6 hpi in the fat body. A previous study also reported similar results in response to NPV that *serpin-28* expression upregulated at 6 hpi [42]. We assume that it may possible that the silkworm immune response activated earlier than virus proliferation. In contrast, *Bmserpin2* expression was upregulated at 48 and 72 hpi in BC9. This showed that *Bmserpin2* expression was delayed until 48 h or 72 h in BC9. This indicates that BC9 resists BmNPV infection more efficiently than P50.

Previous reports have studied the serpin genes in insects and showed increased expression in response to pathogens [22,26,40,43]. Yuan et al. reported that *Helicoverpa armigera* serpins were upregulated after viral infection [32]. Therefore, we assume that induced expression of *Bmserpin2* might also be involved in the regulation of PPO in silkworms after BmNPV infection.

**Figure 11:**
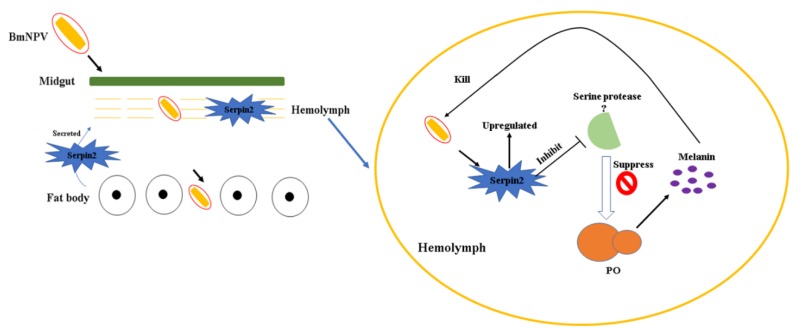
A proposed diagram of role of Bmserpin2 in the regulation of the PO cascade after BmNPV infection.

Melanization is an important immune response in insects. It consists of a cascade of SPs to activate PPO pathway. Serpins act as inhibitor of SPs to regulate the activity of PPO [14]. Previous studies have proven that serpins are involved in the regulation of the PPO cascade [21,24,44]. Several studies have reported that melanization is involved in antiviral immunity [32,45,46]. In *M. sexta*, epidermal growth factor-like motif (Egf) proteins (Egf1.0 and Egf1.5) are small serine proteinase inhibitors that inhibit melanization by regulating PPO [47,48]. Recently, research has shown that melanization is inhibited in silkworms after BmNPV infection [49]. In our study, we found that the *Bmserpin2* response to NPV infection, and PO activity in haemolymph was drastically decreased after BmNPV infection. Yuan et al., also showed that after virus infection PO activity was decreased [32]. However, another study found that PO activity increased in a susceptible strain (871) after infection of BmNPV (BV) [49]. As the ODV and BV infection mechanisms are different, so we assume that this might account for changes in PO activity. After knockdown of *Bmserpin2*, the PO activity was higher than the control (iGFP/NPV). In particular, the viral genomic DNA copies were significantly reduced in *Bmserpin2* depleted haemolymph. Yuan et al. had shown that knockdown of *H. armigera* serpin-9 and serpin-5 increased PO activity, and the virus load was decreased [32]. In our study, *Bmserpin2* showed different expression in P50 and BC9 strains, and knockdown of *Bmserpin2* caused an increase in PO activity. We speculate that BmNPV inhibits host melanization by regulating *Bmserpin2* expression. In the light of these results, we propose a model for the role of *Bmserpin2* in the PO cascade of *B. mori* after BmNPV infection (Figure 11). Once BmNPV penetrates the midgut and enters the fat body, then the fat body will synthesise immune related proteins including *Bmserpin2*, which will be secreted into the haemolymph. In the haemolymph, the PO cascade will be initiated via serine proteases to produce melanin to kill BmNPV. In successful BmNPV proliferation, the virus will overcome the host PO response through upregulating *Bmserpin2*, which eventually will inhibit serine protease to regulate the PO cascade. The aforementioned results manifested that *Bmserpin2* could suppress melanization by regulating PPO after BmNPV infection.

## 5. Conclusions

In conclusion, we have characterised *Bmserpin2* in response to BmNPV infection. *Bmserpin2* expression was significantly up-regulated in different resistant strains after BmNPV infection. PO activity was down-regulated following BmNPV infection and Bmserpin2 showed an inhibition of melanization in the haemolymph. Knockdown of *Bmserpin2* resulted in a dramatic increase in PO activity. However, some questions remain to be answered; for instance, it is not clear which of these SPs is the target of *Bmserpin2* inhibition. Further work is underway in our laboratory to investigate the interaction between the host PPO pathway and BmNPV. These findings provide an insight into the innate immunity in silkworms and its mechanism in response to BmNPV infection.

## Figures and Tables

**Table 1:** The primers used in this study.

Primers	Sequences of Primers (5′ to 3′)	Purpose
*Bmserpin2-F*	CGCGGATCCATGATATTTGCCAGAGTTTTGTT	Protein expression
*Bmserpin2-R*	CCGCTCGAGTCAATTTCGTCCACGGTATT	Protein expression
*Bmserpin2-F*	CAGAATACTTGCTGGCGTTGATA	qRT-PCR
*Bmserpin2-R*	AGCCGAGAATGATGAGCGAAT	qRT-PCR
*BmGAPDH-F*	CATTCCGCGTCCCTGTTGCTAAT	qRT-PCR
*BmGAPDH-R*	GCTGCCTCCTTGACCTTTTGC	qRT-PCR
*GP64-F*	GAAGTAGAAACGCCGCATCG	qRT-PCR
*GP64-R*	GTGGGGTATTCAGGCAGCAGT	qRT-PCR

The restriction enzyme sites are marked in underline.

**Table 2:** Sequences of siRNA against *Bmserpin2*.

Name	Sense Strand	Antisense Strand
siRNA-1	GGAAAUAAAGAACCCUGUUTT	AACAGGGUUCUUUAUUUCCTT
siRNA-2	GCUGCCGAACGAAAUUAAUTT	AUUAAUUUCGUUCGGCAGCTT
siGFP	UUCUCCGAACGUGUCACGUTT	ACGUGACACGUUCGGAGAATT
